# Molecular Motor Proteins and Amyotrophic Lateral Sclerosis

**DOI:** 10.3390/ijms12129057

**Published:** 2011-12-07

**Authors:** Kai Y Soo, Manal Farg, Julie D. Atkin

**Affiliations:** 1 Department of Biochemistry, La Trobe Institute of Molecular Science, La Trobe University, VIC 3086, Australia; E-Mails: k.soo@latrobe.edu.au (K.Y.S.); m.farg@latrobe.edu.au (M.F.); 2 Centre for Neuroscience, University of Melbourne, Parkville, VIC 3010, Australia; 3 Florey Neuroscience Institute, University of Melbourne, Parkville, VIC 3010, Australia

**Keywords:** amyotrophic lateral sclerosis, axonal transport, kinesins, dynein, myosin

## Abstract

Amyotrophic lateral sclerosis (ALS) is a neurodegenerative disorder affecting motor neurons in the brain, brainstem and spinal cord, which is characterized by motor dysfunction, muscle dystrophy and progressive paralysis. Both inherited and sporadic forms of ALS share common pathological features, however, the initial trigger of neurodegeneration remains unknown. Motor neurons are uniquely targeted by ubiquitously expressed proteins in ALS but the reason for this selectively vulnerability is unclear. However motor neurons have unique characteristics such as very long axons, large cell bodies and high energetic metabolism, therefore placing high demands on cellular transport processes. Defects in cellular trafficking are now widely reported in ALS, including dysfunction to the molecular motors dynein and kinesin. Abnormalities to dynein in particular are linked to ALS, and defects in dynein-mediated axonal transport processes have been reported as one of the earliest pathologies in transgenic SOD1 mice. Furthermore, dynein is very highly expressed in neurons and neurons are particularly sensitive to dynein dysfunction. Hence, unravelling cellular transport processes mediated by molecular motor proteins may help shed light on motor neuron loss in ALS.

## 1. Introduction

Amyotrophic lateral sclerosis (ALS), also known as Charcot’s sclerosis or Lou Gehrig’s disease, is the most common form of motor neuron disease. It is characterized by progressive degeneration of motor neurons in the primary cortex, brainstem, and spinal cord, which results in muscle dystrophy, paralysis, and death. The fatal event is usually failure of the respiratory muscles. At least 10% of all ALS cases are inherited (familial ALS); however, most cases have no discernable genetic component (sporadic ALS). Several proteins are linked to sporadic and familial forms of ALS, notably superoxide dismutase 1 (SOD1) [1], TAR DNA binding protein 43 (TDP-43) [2], and fused in sarcoma (FUS) [3]. Both sporadic and familial forms of ALS are clinically and pathologically identical.

Of the cases of familial ALS, approximately 20% are linked to dominantly inherited mutations in SOD1. Mutant SOD1 has been extensively studied in both cellular and animal models of disease. As a multifactorial disorder, several mechanisms have been implicated in the neurodegenerative process in ALS. These include protein misfolding and aggregation, excitotoxicity [4], neuro-inflammation [5,6], endoplasmic reticulum (ER) stress, oxidative stress [7] and mitochondrial dysfunction [8], cytoskeleton abnormalities and defective axonal transport [9,10]. However, the initiating factors which trigger neurodegeneration still remain unknown. Recently, increasing evidence implicates dysfunction of the cellular transport machinery in ALS, including dysfunction to the molecular motor proteins, dynein and kinesin.

A perplexing characteristic of ALS is why motor neurons are targeted by ubiquitously expressed proteins. Motor neurons are large, highly differentiated, polarized cells, with extremely long axons, up to 1 metre in length in an adult human. Hence, they have high synthetic and energy requirements, which therefore places heavy demands on cellular transport processes [11]. Proteins, lipids, mRNA and organelles need to rapidly move from the cell body over large distances along the axon to the synaptic terminals or neuromuscular junctions, where they are required for axoplasmic membrane remodeling, energy production, neurotransmission and local protein synthesis [12]. Axonal transport is also required to collect neurotrophins, survival factors or potentially toxic factors from distal axons, peripheral synapses or muscle cells, back to the soma. The complexity and fine regulation of this system is highly sensitive to perturbation, and minor alterations of cellular and vesicular transport processes may result in motor neuron dysfunction [13].

Axonal transport is a key mechanism required for cellular viability in neuronal cells. Most proteins required in the axon and in synaptic terminals must be transported along the axon after synthesis in the cell body. Similarly RNA and organelles also need to be transported over long distances, and these transport processes require molecular motors, such as kinesins, dyneins and myosins, that operate along the cellular cytoskeleton. Dysfunction of axonal transport has been described in several neurodegenerative diseases, and is well documented in ALS [9,14,15]. In this review, we describe the functions of the molecular motors, including kinesins and dyneins, as well as their putative roles in ALS.

## 2. Genetics of ALS

Although most cases of ALS are sporadic, approximately 10% of all cases are inherited, and investigations into the genetic causes of ALS may prove to be important for uncovering common disease mechanisms [16,17]. Mutations in SOD1 were the first identified and remain the best characterized dominantly inherited causative genetic mutations in ALS [1]. However, in recent years additional genes have been linked with disease and most of these have arisen from studies of small familial pedigrees with dominantly-inherited ALS. However, mutations have also been identified in subsets of sporadic ALS cases, indicating that de novo mutations can cause disease, and suggesting that genetic changes are important in both familial and sporadic ALS.

Mutations in SOD1 are scattered amongst all five exons of the SOD1 gene. SOD1 is a cytoplasmic protein, and mutant SOD1 proteins are prone to aggregation and intracellular inclusion formation. The most widely used and well-characterized experimental model of human ALS is the SOD1^G93A^ transgenic mice, which develops progressive muscular weakness and paralysis similar to human ALS [18]. Structural abnormalities of motor neurons are evident in SOD1^G93A^ transgenic mice, such as dilated ER in axons, dendrites, and cell bodies, as well as vacuoles associated with swollen degenerating mitochondria [19].

The second most common gene linked to ALS after SOD1 is fused in sarcoma (FUS) [3,20] and FUS mutations are found in up to 5% of familial ALS patients [3,20]. Mutations in FUS are found in both familial and sporadic ALS, as well as in patients with ALS and frontotemporal dementia (FTD), and in frontotemporal lobar degeneration (FTLD) patients without motor impairment [3,20–23], suggesting a pathogenic overlap between ALS and other neurodegenerative diseases. Most identified mutations cluster in the C-terminal portion of the protein, around the nuclear localization sequence.

TDP-43 is the major constituent of cytoplasmic and intracellular inclusions in neurons and glia of patients with sporadic and familial ALS [2,24]. More than 30 different mutations in the gene encoding TDP-43, TARDBP, have now been reported in 3 to 4% of cases of familial ALS [25,26]. The functions of both FUS and TDP-43 are largely uncharacterized, although the structural and functional similarities between these two proteins suggest that similar disease mechanisms could operate for both proteins. Transgenic mice overexpressing either wildtype or mutant TDP-43 produces similar phenotypes [27]. However, the mechanisms causing pathogenesis in the mutant TDP-43 transgenic mice remain unknown, although abnormal cytoplasmic mitochondrial aggregates and early lethality have been identified [27].

Several forms of juvenile-onset ALS have been characterized, such as ALS2, with a very slow disease progression. Mutations in ALS2, which are autosomal recessive, are thought to involve vesicle transport and membrane trafficking processes [28]. Another juvenile-onset ALS is autosomal dominant ALS4 linked to mutations in the SETX gene encoding senataxin, which is thought to possess DNA/RNA helicase acitivty [29]. A single mutation, P56S, in vesicle associated membrane protein/synaptobrevin-associated membrane protein B (VAPB) are also described in a form of autosomal-dominant late-onset ALS (ALS8) [30]. Mutations in tau and the p150 dynactin subunit are also described in ALS-dementia and lower ALS respectively, and cause progressive, autosomal dominant forms of diseases and without sensory symptoms [31,32]. In 2010, two further ALS-causative genes, optineurin [33] and valosin containing protein (VCP) [34], were identified. Mutations in optineurin are found in both sporadic and SOD1-linked ALS [33,35]; but VCP mutations have only been identified in familial ALS cases [34]. Mutations in the gene encoding ubiquitin-like protein ubiquilin 2, *UBQLN2*, which cause dominantly inherited, were also recently linked to chromosome-X-linked ALS and ALS/dementia [36]. Ubiquilin 2 is a member of the ubiquilin family, which regulates the degradation of ubiquitinated proteins [36], and mutations in UBQLN2 lead to an impairment of protein degradation [36]. A more recent report identifies a noncoding expanded GGGGCC hexanucleotide repeat in *C9ORF72* as the cause of chromosome 9p-linked FTD/ALS and shows that this genetic defect is the most common cause of ALS and FTD identified to date [37].

## 3. Possible Pathogenic Mechanisms in ALS

Despite the fact that a number of genes have now been linked to ALS, the exact pathogenic mechanisms are still largely unclear. Recent studies have suggested that both sporadic and familial forms of ALS share common pathologic mechanisms [38,39].

### 3.1. Protein Misfolding and Aggregation

Protein misfolding and aggregation are prominent features of ALS, but the relationship to disease pathogenesis remains unclear. A number of different protein inclusions are found in human ALS, and animal models of ALS, including ubiquitinated skein-like inclusions, bunina bodies, and hyaline inclusions rich in neurofilaments proteins [40]. Many proteins have been found within the inclusions in ALS, including neurofilaments proteins and peripherin [41], SOD1, p38MAPK and Cdf4 kinases [42], cystatin C [43], FUS [44] and TDP-43 [2]. SOD1 inclusions have been detected in lower motor neurons of familial ALS patients, mutant SOD1 transgenic mice, and in cultured cells expressing mutant SOD1 [45]. Wildtype SOD1 does not form large intracellular inclusions but may form smaller aggregates under oxidative stress [46]. It is unclear how protein aggregation is toxic to motor neurons, either by sequestration of proteins critical for cell viability, or by interfering with vital cellular mechanisms, such as chaperone activity, inhibiting the ubiquitin-proteasome system and disrupting of cellular transport [16].

### 3.2. Excitotoxicity

Glutamate excitotoxicity results from the excessive influx of calcium cations through the over-stimulation of post-synaptic glutamate receptors, including NMDA and AMPA-type receptors [47]. This increase of calcium can activate enzymes such as phosphatases, proteases, lipases and endonucleases, causing protein and lipid alterations in cell membranes, generation of toxic reactive oxygen species (ROS), and mitochondrial damage and dysfunction [47]. Decreased levels of the excitatory amino acid transporter EAAT2, which is present on astrocytes and is responsible for rapid removal of glutamate from the synapse, are found in both human patients and in mutant SOD1 transgenic rodents [48]. Furthermore, increased glutamate is found in CSF of ALS patients [49]. Riluzole, which is the only approved treatment for ALS at present, is thought to inhibit glutamate release from synapses [50]; however, riluzole only moderately improves survival in ALS patients [51]. Overexpressing EAAT2 in mutant SOD1 transgenic mice decreases excitotoxicity, delays disease onset, slows disease progression and prolongs life-span [52], but only partially increases neuroprotection [53]. Thus, exicitotoxicity may be involved in modulation of disease progression.

### 3.3. Neuroinflammation

Neuroinflammation is characterized in ALS by the appearance of reactive microglial and astroglial cells [5,6], suggesting a non-cell autonomous process [54]. In ALS, reactive astrocytes produce nitric oxide and peroxynitrite, and trigger mitochondrial damage and apoptosis in motor neurons [55]. Astrocytes may also contribute to excitotoxic damage to motor neurons as discussed above. Furthermore, microglial cells are reported to be activated in the brain and spinal cord of patients with ALS, as well as mutant SOD1 transgenic mice [56] and activated microglia were detected before motor neuron loss [56]. Damage within motor neurons is enhanced by injury from microglial cells via an inflammatory response that accelerates disease progression [57]. Furthermore, treatment of ALS with minocycline, a tetracycline derivative that inhibits microglial activation, increases survival and delays disease onset in SOD1^G93A^ transgenic mice [58]. Hence, understanding the cross-talk between motor neurons and their non-neuronal neighbours may lead to understanding how these factors act in concert to drive disease progression.

### 3.4. Mitochondrial Disturbances and Oxidative Stress

Changes in mitochondrial structure and function, and activation of mitochondrial dependent apoptosis, have been described in animal and cellular models of ALS, as well as in patients with ALS [59,60]. In SOD1^G93A^ transgenic mice, mitochondrial degeneration and vacuolation are reported very early, at 2 weeks of age [8,61], suggesting that mitochondrial abnormalities are an early event in disease pathogenesis. Furthermore, mutant SOD1 associates with mitochondrial compartment, such as intermembrane space of mitochondria, from where it may trigger apoptosis [62]. Mitochondrial-dependent apoptosis, including redistribution of cytochrome c from mitochondria to cytosol and recruitment of proapoptotic Bax to mitochondria are associated with mutant SOD1 inclusion formation [60]. Dysfunction of components of the mitochondrial respiratory chain are also evident in the spinal cord of SOD1^G93A^ transgenic mice at disease end stage [63]. Expression of mutant SOD1 in cell culture induces a significant loss of mitochondrial membrane potential and increases mitochondrial ROS production, suggesting that respiratory chain dysfunction and mitochondrial depolarization are underway [7]. Impaired axonal transport of mitochondria has been reported in ALS [64], although a recent study demonstrates that impairment of mitochondrial transport in SOD1^G93A^ transgenic mice plays a minimal role in ALS pathology [65]. Furthermore, mutant SOD1 targeting to the mitochondria impaires mitochondrial dynamics and also is associated with impaired maintenance of neuritic processes [66]. Axonal transport of mitochondria may be essential to neurons due to their extensive processes between the cell body and the synapse at the cell periphery [67].

### 3.5. ER Stress

We and other groups have demonstrated that ER stress is an important pathway to cell death in ALS [68,69], and is triggered very early in SOD1^G93A^ transgenic mice at p5 [70]. ER stress is triggered when misfolded proteins accumulate within the ER lumen, inducing the unfolded protein response (UPR). Although the initial phases of the UPR aim to promote cell survival, prolonged or severe ER stress triggers the apoptotic phase of the UPR. Upregulation of the three UPR sensor proteins, PERK, ATF6 and IRE1, have been observed both at the symptom onset and at disease end stage of SOD1^G93A^ transgenic mice, implying the involvement of ER stress in disease mechanisms [68,71]. The ER chaperone, protein disulphide isomerase (PDI), was found to co-localize with mutant SOD1 inclusions in both cellular and animal models of ALS [72] and overexpression of PDI decreased mutant SOD1 aggregation, ER stress, and apoptosis [72]. However, it remains unclear how ER stress is triggered in ALS because SOD1 and the other proteins linked to ALS are not associated with the ER.

## 4. Axonal Transport in Neurons

The major roles of axonal transport are to move newly synthesized proteins or lipids to the distal axon or synapse to maintain axonal and synaptic activity, and to clear proteins and organelles from the axon or synapse to the cell soma for efficient degradation. Axonal transport also plays a role in the communication of intracellular signals from the distal axon to the soma, allowing the neuron to respond to changes in the environment. Another important function is the transport of mitochondria to provide energy in the axon or synapse. Defects in either supply or clearance of material within an axon can lead to neuronal death.

Molecular motors from the kinesin, dynein, and myosin super-families transport cargo within neurons and other cell types [73,74]. Both microtubules and neurofilaments are the major longitudinal cytoskeletal filaments within the axon and dendrites and the molecular motors move along microtubules during transport. In the synaptic regions, such as pre-synaptic terminals and post-synaptic spines, actin filaments form the main cytoskeletal architecture [75], and myosins are the main molecular motor which conveys cargos along actin filaments [76]. Axonal transport occurs bi-directionally, from the cell body to the periphery (anterograde transport) and from the periphery to the cell body (retrograde transport). The direction of transport depends on the polarity of the rails. In the axon, microtubules have unipolar orientation: the fast growing “plus” end points towards the synapse, and the “minus” end points towards the soma [77]. However, in the dendrites, the microtubule polarity is mixed. Actin filaments also have polarity: the barbed end (the growing end) points to the plasma membrane in pre-synaptic and post-synaptic regions.

Classically and functionally, axonal transport is divided into fast and slow axonal transport based on the bulk speed of cargo movement. Vesicles and mitochondria move by fast axonal transport at speeds of ~1 μm/s, whereas cargoes such as cytoskeleton components move slowly at speeds of ~1 mm/day [78]. Movement is also intermittent in slow axonal transport with individual cargoes pausing during their transit within the axon [15,79].

## 5. Molecular Motor Proteins: Kinesin, Dynein, Myosin

Kinesin superfamily proteins (KIFs) are composed of a motor domain, a stalk domain and a tail region [80]. The conserved globular motor domain consists of an ATP-binding site and a microtubule-binding sequence. The motor domain hydrolyzes ATP and transfers chemical energy for the movement of each KIF along microtubules with intrinsic directionality. The tail regions and the filamentous stalk regions recognize and bind to the cargo(s) [81]. While motor domains show high amino acid sequence homologies (30–60%) among the KIFs, the other regions exhibit significant variability [82,83].

KIFs can be broadly grouped into three types depending on the position of the motor domain within the molecule. The motor domain is found in the NH_2_-terminal region in N-kinesins, in the middle of the protein in M-kinesins, and at the COOH terminus in the C-kinesins. N-kinesins generally move toward microtubule plus ends, while C-kinesins move toward minus ends, and M-kinesins depolymerise microtubules [84,85]. The kinesin superfamily comprises at least 45 members in mammals such as human and mouse [86] and the KIFs are classified into 14 families based on head domain homology [86–89]. These include one M-kinesin family, one C-kinesin family, and 12 N-kinesin families. KIFs usually drive anterograde transport while most retrograde transport is mediated by cytoplasmic dynein, although a few kinesins also power retrograde transport [90–92].

In striking contrast to the kinesin family with its diverse members, cytoplasmic dynein utilizes its single structural form to carry out a wide variety of cellular functions, many of which are similar to those produced by the kinesins. Dyneins are members of the AAA+ superfamily (AAA), ATPase-associated proteins with various cellular activities [93]. Dynein motor proteins couple ATP hydrolysis to movement along microtubules towards the minus end and they can carry a large variety of cargoes. Dynein superfamily proteins comprise two major groups, cytoplasmic dynein and axonemal dyneins, which are also called ciliary or flagellar dyneins. The complete dynein motor is a large, multi-protein complex (1.5 MDa) composed of two identical heavy chains, two intermediate chains, four light intermediate chains and several light chains. Cytoplasmic dynein has an important associated protein complex called dynactin, containing p150^Glued^, p62, dynamitin, actin related protein (Arp) 1, CAPZα and CAPZβ, p27, and p24. Dynactin regulates dynein activity and the binding capacity of dynein for its cargos [94]. The homodimer heavy chain of dynein binds to microtubules and enables dynein to move in an ATP-dependent manner [95]. The other subunits of dynein are thought to maintain the stability of the complex, to modulate its activity and to interact with accessory and cargo proteins [96–98]. Cytoplasmic dynein may also perform tasks other than the transport of cargos; for example, endosomes depend on dynein not just for their motility, but also for their maturation, morphology and receptor sorting [99].

Dynein and kinesins function in an inter-dependent manner, and the disruption of movement in one direction can also affect movement in the opposite direction [100]. The intermediate chain of dynein can bind directly to kinesin light chains 1 and 2 [101], and dynactin is able to interact not only with dynein but also with several kinesins [102]. Hence, the regulation of axonal transport processes extremely complex.

Myosin superfamily motor proteins bind to actin and hydrolyse ATP to generate force and movement along actin filaments. They are classified into 18 classes, mostly based on comparisons and phylogenetic analysis of the conserved motor domain [103]. They play significant roles in cell movement, muscle contraction, cytokinesis, membrane trafficking, and signal transduction. Most myosins form a dimer and consist of a motor domain, a neck region, and a tail region.

## 6. Molecular Motors in SOD1-Associated ALS

There is increasing evidence for dysfunction of axonal transport in the pathogenesis of ALS. In SOD1^G93A^ transgenic mice, defects in both anterograde and retrograde axonal transport are observed [104]. There is also evidence that impairment of axonal retrograde transport is one of the earliest axonal pathologies in SOD1^G93A^ transgenic mice, suggesting deficits in axonal transport are a key pathogenic event in ALS [10]. Both fast and slow axonal transport were also impaired in the ventral roots of transgenic mice with low copy number of mutant SOD1 [105]. Swollen axon segments or spheroids are also present in spinal cords of human ALS and SOD1^G93A^ transgenic mice [106]. Also, axonal disorganization and the accumulation of neurofilament proteins are hallmarks of both sporadic and inherited forms of ALS [107–109]. Mice overexpressing human neurofilament heavy-subunit gene (NF-H) posses dramatic defects in axonal transport, not only of neurofilament proteins but also of other proteins, including tubulin and actin [107]. Point mutation of the p150 subunit of dynactin has been detected in both sporadic and familial ALS patients [110]. Mutations in dynactin lead to a reduction in retrograde transport but disease progression is less severe than ALS in transgenic mice [31]. In contrast, mutations in cytoplasmic dynein can either result in pure sensory neuropathy or in a sensory neuropathy with motor neuron involvement [111]. A recent study suggested that alterations in retrograde signalling contribute to neurodegeneration in ALS, by shifting from the transport of survival-promoting to death-promoting signalling cargo molecules [112]. Mutant SOD1 also inhibits anterograde fast axonal transport of mitochondria enhances the retrograde movement resulting in a depletion of axonal mitochondria content [113]. Mislocalization of mutant SOD1 is observed in axonal mitochondria within motor neurons, accompanied by intra-axonal accumulation of misfolded SOD1 [114]. Considering the very long axon of the motor neuron and the relative abundance of mitochondria within these axons, mitochondrial transport disturbances are expected to have a significant impact on the overall function of the motor neuron [114]. Evidence suggests that axonal transport alterations are an early event in ALS, thus implicating axonal transport in the pathogenesis of ALS.

### 6.1. Kinesins

Kinesins mediate anterograde axonal transport, which is essential for synapse generation and for maintaining synaptic transmission [80]. Several studies have highlighted the importance of kinesin in ALS, particularly kinesin heavy chain genes, KIF5A and KIF1Bβ, which transport mitochondria, synaptic vesicles and macromolecular complexes. A recent study demonstrated that oxidized wildtype SOD1, and wildtype SOD1 immunopurified from sporadic ALS patient tissues, inhibited kinesin-based fast axonal transport in a manner similar to mutant SOD1 in familial ALS, suggesting common pathogenic mechanisms in both sporadic and familial ALS [38]. Disruption of KIF5A in neurons postnatally does not affect fast axonal transport; however, it induces a reduction in slow anterograde axonal transport, resulting in an accumulation of neurofilament proteins in the cell bodies of peripheral sensory neurons and a reduction in sensory axon calibre [115]. Similarly, the expression of KIF1Bβ mutants in mice induces defects in the anterograde transport of synaptic vesicle precursors, resulting in a late-onset axonopathy [116]. These pathological effects closely mimic those observed in individuals bearing mutations in the KIF5A and KIF1Bβ genes, which have been identified in families with hereditary spastic paraplegia [117] and Charcot-Marie Tooth type 2A neuropathy [116]. Furthermore, not only mutations, but lower expression of KIF has been associated with neurodegeneration. For example, reduction in the levels of kinesin associated protein 3 (KIFAP3) has been linked to increased survival in ALS patients [118], which may be due to changes in axonal transport of choline acetyltransferase (ChAT) [119]. Hence, KIFAP3 can be considered as a potential modifier of the ALS phenotype [120]. KIFAP3 is part of the trimeric motor kinesin II complex (KIF3) that is involved in multiple functions including intracellular transport [121]. Furthermore, a specific downregulation of kinesin-related proteins, KIF1Bβ and KIF3Aβ, has been detected in motor cortex specimens of sporadic ALS patients [122]. Nevertheless, interaction between mutant SOD1 and members of the kinesin-1 family (KIF5A, 5B, or 5C) was not observed in spinal cords of SOD1^G93A^ transgenic mice at 60 days of age, suggesting that impairment of anterograde transport occurs on the verge of developing clinical symptoms [123]. In comparison to dynein, relatively few degenerative diseases have been directly linked to kinesin, possibly due to functional redundancy in the large kinesin superfamily.

### 6.2. Dyneins

Dynein is very highly expressed in neurons [124] and neurons are particularly sensitive to defects in dynein/dynactin function [78]. A dynein subunit was identified as a component of mutant SOD1-containing high molecular weight complexes prior to the onset of symptoms in ALS animal models and increased during disease progression [125]. Furthermore, mutations in the dynactin 1 gene (DCTN1), encoding the p150Glued subunit of dynactin, cause human distal hereditary motor neuropathy (HMN7B) [31,126]. Knock-in and transgenic mice expressing these mutations subsequently develop motor neuron degeneration [127,128]. Over-expression of the dynactin subunit dynamitin in mice leads to impairment of dynein-dynactin interaction and a late-onset progressive disease reminiscent of ALS [129]. Mutation of the dynein heavy chain 1 gene causes motor neuron degeneration and inhibition of axonal transport [130]. *Loa* (legs at odd angles) and *Cra1* (Cramping1) mice which carry separate N-ethyl-N-nitrosourea-induced mutations of the dynein heavy chain 1 gene, show selective impairment of axonal retrograde transport as well as progressive locomotor disorders associated with spinal motor neuron degeneration [130]. Also, a third mouse model (*Swl*) with another dynein heavy chain gene mutation displays an early-onset sensory neuropathy with muscle spindle deficiency [131]. Hence, the relative contributions of sensory and motor neuron degeneration to the phenotypes of *Loa*, *Cra1*, and *Swl* mice are still under debate.

Surprisingly, crossing the SOD1^G93A^ transgenic mouse with *Loa* or *Cra1* animals delays disease progression and significantly increases lifespan [132]. This amelioration correlates with a complete recovery of the axonal transport deficits in motor neurons of these mice [132,133]. However, this observation was partially explained recently by a proteomics study which revealed that the SOD1 mutation augments retrograde transport of stress factors (p-JNK, caspase-8 and p75NTR cleavage fragment) and simultaneously impairs retrograde transport of survival factors (p-Trk and p-Erk1/2) [134]. Dynein also plays a role in the transport of nerve injury signals, phosphorylated Erk [135], and phosphorylated JNK [136]. Many types of protein cargoes transported by dynein are implicated in ALS [134]. Impaired retrograde transport of activated neurotrophin receptors by direct association of the dynein light chain with Trk neurotrophin receptors may also trigger neurodegeneration [137,138]. Dynein is involved in the autophagic clearance of misfolded proteins [139] and removal of damaged organelles and proteins from the axonal compartment [140]. Therefore, clearance of misfolded proteins in ALS is affected by dysfunction of dynein.

It is clear that dynein-mediated retrograde axonal transport is affected in motor neurons in ALS [123]; however, the underlying mechanisms are still unclear. Mutations in the dynein-dynactin machinery cause phenotypes that may influence multiple processes including neurotrophic factor delivery, transport, and homeostasis of mitochondria and protein aggregation or degradation. Disruption of dynein function could therefore link several proposed pathways relevant to ALS pathophysiology, including axonal transport, mitochondrial dysfunction, and mutant SOD1 aggregation.

## 7. Dynein-Mediated ER-Golgi Transport in ALS

The transport of secretory proteins between the ER and Golgi apparatus is also dynein mediated, and dysfunction of the ER-Golgi is also implicated in diseases of motor neurons, suggesting a link between ER-Golgi and axonal transport processes [129]. Also, dynein dysfunction can result in fragmentation of the Golgi apparatus [141], which occurs in spinal and cortical motor neurons in ALS patients, in cell lines over-expressing mutant SOD1 [142], and is also one of the first pathological events in SOD1^G93A^ transgenic mice [143].

The ER and Golgi apparatus regulate protein trafficking and secretion. The ER is responsible for the sorting, post-translational modification and trafficking of transmembrane and secretory proteins [144,145]. Vesicles containing secretory protein cargo bud from the ER, fuse with the cis-Golgi network and subsequently progress to the trans-Golgi network, from where they are transported to further cell was locations. Approximately a third of all cellular proteins are secreted via this classical ER-Golgi pathway. Hence, inhibition of ER-Golgi function and transport could severely impact on cellular function. The relationship between dynein-mediated ER-Golgi transport and axonal transport mechanisms is poorly understood, but the two processes are clearly linked (recently reviewed in [146]).

### 7.1. ER-Golgi and Diseases of Motor Neurons

Protein folding in the ER is required to maintain cellular function. Protein misfolding within the ER induces ER stress, triggering the UPR. The UPR mechanisms aim to restore homeostasis, but if homeostasis is not restored, cell death is triggered via apoptosis [147]. Cellular insults which lead to increased protein misfolding in the ER include changes in intracellular calcium concentration, alterations in the redox state of the ER, nutrient deprivation, failure of post-translational modifications and increases in secretory protein synthesis [148]. ER stress also induces ER-associated degradation (ERAD), a process by which proteins are removed from the ER and retrotranslocated to the cytosol, where they are degraded by the 26S proteasome. It has been reported that UPR induction is due to the interaction of cytoplasmic mutant SOD1 with Derlin-1, a key protein involved in ERAD [149], but this interaction was only detected after the onset of symptoms in transgenic SOD1 mice, suggesting that this mechanism is not the primary trigger of ER stress. However, it is now recognised that other cellular processes can cause secondary ER stress, such as disturbances of the ER, Golgi, endosomal and vesicular transport systems [150–152].

ER stress has been reported as a key role in mutant SOD1-linked ALS pathogenesis; however, the exact mechanisms that trigger ER stress in ALS remain elusive. Recent evidence also implicates the ER-Golgi in disease induced by TDP-43 and FUS in ALS. Recently, TDP-43 was detected in the rough ER in spinal cord motor neurons of sporadic ALS patients [153], suggesting that TDP-43 could be redistributed to the ER in disease. Furthermore, TDP-43 has been detected in mouse brainstem microsomes which contain ER and other vesicles [154], and pharmacological induction of ER stress increases the accumulation of TDP-43 in cell culture [155]. Similarly, FUS has also been linked to the ER: electron microscopy of spinal motor neurons of patients with juvenile ALS demonstrated FUS-positive inclusions associated with disorganised rough ER [156]. A key UPR protein, BiP, is also found in FUS-immunoreactive inclusions in ALS patient tissue, providing further evidence that ER stress could be involved in mutant FUS-linked ALS [157].

### 7.2. ER-Golgi Vesicle Trafficking Defects

Vesicle trafficking defects have been linked to human motor neuron diseases [158]. Dynamin 2, a vesicular traffic regulator modulates the actin cytoskeleton, interacts with actin-binding proteins such as profilin and Abp1 [159] and is involved in the fusion and fission of vesicles and other membranous organelles [160]. Atlastin 1 (SPG3A) and REEP1 mutations (SPG31) also result in marked ER morphological defects and altered synaptic vesicle recycling. Several familial forms of ALS are also linked to genes encoding proteins involved in the regulation and control of vesicle transport: VAPB, VCP and ALS2 [34,161,162]. Mouse mutants with motor phenotypes have been linked to defects in intracellular trafficking, such as the progressive motor neuronopathy (pmn) mouse, which accumulates the mutant tubulin-specific chaperone TBCE in the cis-Golgi compartment [163]. Also, the wobbler mouse, which displays motor neuron dysfunction, contains mutations in vacuolar-vesicular protein sorting factor Vps54, which is involved in Golgi-associated vesicular trafficking [164].

#### (a) Vesicle-associated membrane protein-associated protein B (VAPB)

Missense mutations of VAPB, a widely expressed ER transmembrane protein [165], cause autosomal dominant typical ALS, slowly progressing atypical ALS or late-onset spinal muscular atrophy (SMA). VAPB is a highly conserved protein, which is localized in the ER and associated with microtubules [166]. VAPB has important function in ER and Golgi maintenance and neuronal transmission [165,167,168]. It is also involved in the regulation of ER−Golgi vesicle transport [169] and in modulating ER stress [170]. The two VAPB mutations described in ALS, P56S and T46I, perturb ER and Golgi trafficking [171] and cause morphological disruption to the ER [168,172,173]. These mutations also appear to increase motor neuron vulnerability to ER stress-induced death, by sequestering wild-type VAPB [174]. Mutant VAPB also induces the co-aggregation of wild-type VAPB [170,173], suggesting a dominant-negative mode of pathogenesis. Mutant P56S VAPB inclusions are ER derived [165,168], and co-locate with a subset of ER proteins [172]. VAPB proteins are also involved in axonal guidance [175], which may lead to axonal dysfunctions and contribute to ALS [172].

#### (b) Optineurin

Optineurin is also one of the disease-causing genes in ALS although it was earlier reported to be a causative gene in primary open-angle glaucoma. Optineurin is a Golgi-localized protein [176,177]; however, mutations in optineurin induce the formation of cytoplasmic inclusions in both sporadic and SOD1 cases of ALS [33,35]. Several interacting partners of optineurin have been recently identified, including GTPase molecular Rab8 [178], myosin VI [179], and transferrin receptor [180]. The redistribution of optineurin from the Golgi apparatus to the cytoplasm leads to Golgi fragmentation [181]. Together these data indicate a possible role for optineurin in protein trafficking and axonal transport dysfunction [182].

#### (c) Valosin containing protein (VCP)

Recently using exome sequencing, ALS-causative mutations in the gene encoding valosin containing protein (VCP/p97) were identified in familial ALS patients [34]. VCP is a hexameric type II ATPase of the AAA family, with many putative roles, including transcription, cell division, Golgi assembly, autophagy, and in particular, aiding proteasome function [183–186]. Knockdown of VCP in HeLa cells leads to an increase in ubiquitinated proteins derived from the ER [187], and reduced levels of cellular VCP induce ER stress, perhaps as a consequence of reduced ERAD and/or by disturbing the fusion of ER membranes [188]. Motor neurons of an ALS patient with a VCP mutation were recently found to contain TDP-43-positive inclusions [34], similar to VCP mutations linked to frontotemporal lobar dementia (FTLD) [189]. VCP mutations in FTLD were also found to induce ER stress and cell death in cell culture [189]. Interestingly, VCP is involved in ERAD by binding to the ER transmembrane protein Derlin-1 [190,191]. The association of Derlin-1 with activation of the UPR in mutant SOD1 cellular and mouse models of ALS [192] suggests overlap in the pathogenic disease mechanisms triggered by VCP, TDP-43 and SOD1 related to ER stress and ERAD.

## 8. Do Mutant TDP-43 and Mutant FUS also Induce Molecular Motor Dysfunction?

Most of the evidence demonstrating the involvement of molecular motor proteins in ALS comes from the study of SOD1 disease models. A role for the other major proteins recently linked to ALS, TDP-43 and FUS, in molecular motor dysfunction remains yet to be established. Translocation of both TDP-43 and FUS from the nucleus to cytoplasm induces the formation of inclusions and is linked to pathology [193–195]. Previous studies have shown that axonal ligation induces transient redistribution of TDP-43 to the cytoplasm and peripheral axonal accumulation of TDP-43 in brainstem motor neurons [154,193]. Moreover, nuclear-excluded TDP-43 co-localized with RNA transport markers and stress granules, indicating that TDP-43 plays a role in sequestering and regulating mRNA levels in response to axonal injury [154]. Both TDP-43 and FUS also play a role in mRNA transport to dendrites in an activity-dependent manner [196]. These data together suggest that accumulation of TDP-43 or FUS in the cytoplasm could lead to transport impairment and axonal injury. Due to the functional similarities between TDP-43 and FUS, the pathological mechanisms associated with these two ALS-linked proteins may be overlapping.

## 9. Conclusions

Motor neurons are highly polarized cells, and hence cellular transport is a fundamental process for the survival and maintenance of motor neurons. Increasing evidence implicates transport defects in the etiology of ALS. Studies using SOD1 transgenic mice clearly show that axonal transport impairment contributes to motor neuron degeneration. Moreover, mutations in genes encoding motor proteins provide direct evidence for a role in the pathogenesis of ALS. Although dysfunction of motor proteins in ALS directly triggers defects in transport, it is possible that other ALS-associated proteins are directly or indirectly linked to an impairment of transport. However, this requires further studies. Hence, identifying the common pathophysiological mechanisms in ALS is crucial. A powerful combination of genetic, cellular, and biochemical approaches have revealed new and often unexpected insights, and the further development of our understanding of molecular motors and their role in ALS will hopefully lead to the development of improved therapies for ALS.
